# Sex Differences in Astrocyte Activity

**DOI:** 10.3390/cells13201724

**Published:** 2024-10-18

**Authors:** Elisa Gozlan, Yarden Lewit-Cohen, Dan Frenkel

**Affiliations:** 1 Department of Neurobiology, George S. Wise Faculty of Life Sciences, Tel Aviv University, Tel Aviv 6997801, Israel; elisag@mail.tau.ac.il (E.G.); lewitcohen@mail.tau.ac.il (Y.L.-C.); 2 Sagol School of Neuroscience, Tel Aviv University, Tel Aviv 6997801, Israel

**Keywords:** astrocytes, sex, neurological diseases, senescence, aging

## Abstract

Astrocytes are essential for maintaining brain homeostasis. Alterations in their activity have been associated with various brain pathologies. Sex differences were reported to affect astrocyte development and activity, and even susceptibility to different neurodegenerative diseases. This review aims to summarize the current knowledge on the effects of sex on astrocyte activity in health and disease.

## 1. Introduction

The first astrocyte-like cell found in the phylogenetic tree was the proto-astrocyte found in nematodes. These proto-astrocytes maintained neural homeostasis and developmental functions of the nervous system but did not cause neuronal death when removed [1]. Later in evolution, astrocyte abundance and complexity skyrocketed and was deemed imperative to neuronal survival; in contrast, the neuronal morphology and function remained surprisingly conserved. Astrocytes have made significant evolutionary progress, exemplified by the differences that arose overtime in the glia-neuron (GNR) ratio between early simple-brained creatures and advanced humans [2,3]. For example, the GNR in *C. elegans* stands at 0.18, whereas the isotropic fractionator published a human GNR of 1, underlining the growing reliance neurons have developed on glial cells [2,3]. However, the exact GNR ratio is still being researched and depends on the brain region, although the major understanding in the field points to an increase in complexity of glia activity rather than an increase in the amount of glial cells, among them astrocytes [4,5,6]. Indeed, several studies insinuate that the human brain has become increasingly dependent on astrocytes to achieve some of its high-level tasks. For example, astrocytes play a crucial role in synaptic connectivity between neurons [7,8]. Based on rodent research, it was suggested that the final maturation of astrocytes happens between three and five weeks [9]. The full maturation process in humans is yet to be discovered [10,11]. While it was previously shown that there are significant changes affiliated with the astrocyte morphology and activity in different brain regions, there are not yet specific cell surface markers that can differ between these populations. Santiago Ramón y Cajal was the first to differentiate between the two main types of astrocytes that were found in the gray matter versus the white matter [12]. He compared the protoplasmic astrocytes with short- and long-branch organelles in the gray matter to the fibrous astrocytes with long unbranched organelles in the white matter. Indeed, the histologic assessment of the human brain based on the Glial Fibrillary Acidic Protein (GFAP) revealed that there are at least ten different morphologies of astrocytes [13]. This finding was confirmed by RNA-seq studies [14]. Furthermore, the GFAP may not be expressed in all astrocytes, while other astrocytic markers such as Aldehyde Dehydrogenase 1 family member L1 (ALDH1L1) and S100 Calcium-Binding Protein B (S100B) can be found in GFAP-/- astrocytes in different brain regions. Thus, there may potentially be an even higher number of astrocytes than reported [14]. Moreover, the complexity of their morphology changes over time, making them one of the most affected and intriguing cells in the brain [15,16]. For example, human astrocytes have evolved to a volume that is 27 times greater than rodents [15].

Astrocytes are important for synaptic plasticity and are suggested to play a role in learning and memory [17]. They form interactions with other brain cells through their processes and regulate brain activities, from synaptic transmission to controlling the blood–brain barrier (BBB) [18]. The integrity of astrocytes is crucial for normal brain development, as strongly demonstrated in “Alexander’s disease”, a neurodegenerative disease affiliated with a missense mutation in the *GFAP* gene, which can lead to brain dystrophy [19]. Astrocytes are sensitive to any changes in the brain milieu and respond accordingly to pathological situations [20]. The dysfunction of astrocyte homeostatic mechanisms is proposed to play a role in neurodegeneration [20]. Astrocytes were reported to undergo significant changes during aging, including morphological alterations and a reactive state characterized by releasing inflammatory mediators. This phenomenon may be due to age-driven cognitive decline or perhaps to the age-driven progression of neurodegenerative diseases [21]. The progression and evolution of the astrocytes have been explored in recent years, considering their critical impacts on neural processes, but little light has been shed on the sexual dimorphism in astrocytes. With astrocytes from both sexes bearing all estrogen receptors and regulating neuroendocrine pathways and factors, several examples of sexually dimorphic astrocytic abilities have been recorded. Astrocytic involvement and responses to neurological pathologies, of which affect males and females in a biased manner, vary greatly. Astrocytes have underlying sex differences, which could bear an impact on their abilities to respond to brain trauma, injury, or pathogenic attacks [22]. Further complexity in astrocyte activity might be seen through these sex differences (Table 1).

## 2. Sex Differences in Astrocytic Differentiation

It was suggested that either the X-chromosome dosage and/or testosterone levels, depending on the region in the brain, result in differing brain structures, function, and gene expression in males and females [45,46,47,48]. Therefore, a certain sexual dimorphism can be expected in astrocytes due to gonadal steroid hormones known regulatory roles in these cells. The amount of astrocytes between males and females in the brain as a whole does not differ, but sexual dimorphism can be found in certain regions in both number and morphology [46,47].

**Figure 1:**
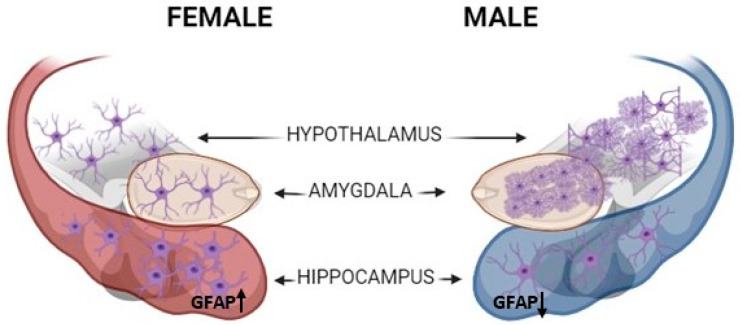
Sexual differences in astrocyte morphology in murine MePD, hypothalamus, and hippocampus. MePD and Hypothalamus: Males contain more in number and more complex astrocytes than females. Hypothalamic male astrocytes are also more differentiated than females. Hippocampus: emales contain more astrocytes, though with smaller processes, and express higher GFAP+ levels than males. Created with BioRender.com.

Differences in astrocyte morphology have been reported between sexes. Morphological sex differences can already be seen in murine astrocytes at the neonatal stage. Male astrocytes were reported to reach full maturation faster than females [38] and to express higher GFAP levels than female astrocytes [49]. It was found that male rat neonatal astrocytes are more differentiated and have significantly longer processes than female astrocytes. Furthermore, the injection of testosterone or estrogen into females or males, correspondingly, could abolish sex differences in branch profile and length. This finding emphasizes the important role of the steroidal impact on astrocytic morphological differentiation [37]. The right posterodorsal medial amygdala (MePD) is rich with gonadal receptors and thus constitutes a most sexually dimorphic area. In rodents, this area in males contains a significantly higher number of astrocytes than in females. Furthermore, male astrocyte complexity in this region is greater than in females regarding the number of branches and their length. Nevertheless, the surface density in female astrocytes is bigger than in male astrocytes [42] (see Figure 1). It was reported that the hippocampus, specifically the CA1 and dentate gyrus (DG) regions, consists of more astrocytes in female mice than males, although female processes were smaller (see Figure 1) [26]. In addition, a study performed on diencephalic cells isolated at different ages found a significantly higher amount of GFAP+ cells in developing female rats as compared to males [25,50]. Thus, while gonadal hormones may account for some sexual variance in astrocytes, other factors must have an impact on these disparities as well.

## 3. Sex Differences in Gene Expression

Sexual dimorphism in the gene expression of astrocytes was initially overlooked, with gene-based sex bias found primarily in microglia (264 genes), then neurons (69 genes), and only lastly, astrocytes, with only 30 sexually dimorphic genes in neonatal mice [36]. Interestingly, a recent study on human cortical astrocytes proves otherwise, representing the only published research on sexual dimorphism in astrocytes in the human species. In the analysis, Krawczyk et al. (2021) found 105 genes that have significantly different levels of gene expression between male and female astrocytes in humans [35]. For example, Zinc Finger Transcription Factor (ZFX) expression is significantly higher in female astrocytes compared to males. This transcription factor has been proven to contribute to the accumulation of the hyperphosphorylated tau protein, which leads to the formation of neurofibrillary tangles, a pathological hallmark of Alzheimer’s Disease (AD), a widely recognized sexually biased disease that impacts women 2-folds more than men [35,51]. Lysine demethylases *KDM6A* and *KDM5C* are also over-expressed in female astrocytes compared to their male counterparts. The lower expression of demethylases in males puts them at a greater risk for mutations which lead to their impairment, which has been linked to a higher risk of developing an Intellectual Disability (ID). This disorder is indeed more common in males than in females. Furthermore, such transcription factors and epigenetic modifications have been seen in the natural aging process and contribute to the development of neurodegenerative diseases [52]. Astrocytic G-protein-Coupled Receptors (GPCRs) take part in cognitive and behavioral signaling, including metabotropic glutamate receptor 3, or *mGluR3*, a known glutamate receptor abundant in hippocampal and cortical astrocytes. It was found that reduced levels of *mGluR3* affected females more than males in memory loss [27]. The glutamate uptake by astrocytes prevents glutamate excitotoxicity, thereby regulating synaptic communication, and acts one of the most vital metabolic roles of astrocytes in the brain [53]. Additionally, astrocytes store glycogen and enable ATP production in neurons via astrocyte-derived lactate. Although astrocytes favor the glycolytic route for energy, mitochondrial oxidative phosphorylation in astrocytes regulates astrocyte reactivity and breaks down fatty acids [54]. Some of these metabolic behaviors in astrocytes also seem to be sexually imbalanced. For example, murine male astrocytes have a higher mitochondrial maximal respiration rate, meaning a larger respiratory capacity to complete oxidative phosphorylation in the case of a high energy demand [39]. Mixed results have been found regarding differing levels of the synaptic density between the sexes, which have led to the finding that murine male astrocytes support the generation of more synapses than females when exposed to certain astrocyte-secreted factors such as thrombospondin-2 (TSP2) [40,55]. Interestingly, TSP2 promotes synaptogenesis via estrogen-mediated pathways, and still, its activity is significantly increased in male astrocytes [40].

## 4. Influence of Estrogen on Sex Differences in Astrocytes

Both male and female human astrocytes express estrogen receptors (ER), including ERα, ERβ, and G protein-coupled estrogen receptor 1 (GPER), as was shown in the cell culture and post-mortem brain tissue [22]. Even so, due to its higher levels in females, estrogen contributes to sex differences in astrocytes via modifications of gene expression and ultimately results in differential activity between males and females. For example, hypothalamic astrocytes in female rats produce progesterone in response to estrogen, while male astrocytes are unable to do so because of the lack of high luteinizing hormone (LH) levels. [56]. Female astrocytes appear more responsive to estrogen than male astrocytes, likely due to the higher expression of estrogen receptors [57]. Genome-wide analyses of gene expression in hippocampal astrocytes from midlife female mice with the selective deletion of ERβ in astrocytes revealed the dysregulation of genes involved in the glucose metabolism as compared to male astrocytes [28].

Sex hormones directly affect the astrocytic response to neuropathological states as they easily traverse the BBB due to their lipophilicity and compactness [58]. Estradiol (E2), the most potent form of estrogen in the body, plays a significant role in neural homeostasis [59]. In addition to regulating the blood flow, mood, and neurological functions, E2 in the brain is most strongly associated with neuroprotection [60]. Astrocytes have the unique ability to internalize E2 by expressing estrogen receptors and advancing a myriad of cascades, including activating astrogliosis in response to neuroinflammation and modulating aquaporin 4 (AQP4) expression levels which control edema, glutamate transporters, and even intracellular Ca^2+^ levels [22,61,62]. In addition to gonadal E2, astrocytic-derived E2 activates astrocytes in response to brain trauma to secrete neurotrophic factors and clear neurotoxic glutamate as a compensatory mechanism for retention of neuronal viability [63,64,65].

The intertwined estrogen–astrocyte relationship may influence the sex differences observed in different brain diseases, probably due to higher estrogen levels in females [57]. In animal models, estrogen may alter the astrocyte morphology to a more ramified structure. This could affect their interactions with neurons and other cells in the brain [66]. Estrogen promotes neuronal sustainability indirectly by enhancing astrocytic glucose uptake and metabolism [22]. Additionally, estrogen can modulate calcium signaling in astrocytes, which is especially essential for gliotransmitter release. E2 stimulates astrocytes to produce growth factors such as brain-derived neurotrophic factor (BDNF) and insulin-like growth factor-1 (IGF-1), which support neuronal survival and plasticity [22]. Additionally, estrogen has been shown to increase the expression of glutamate transporters in astrocytes. This enhances their ability to remove excess glutamate from the synaptic cleft, thereby reducing excitotoxicity [67]. Of note, many of these differences become more pronounced after puberty, coinciding with increased estrogen levels in females. Indeed, estrogen treatment can partially attenuate sexual dissimilarities in astrocyte activity when administered to male astrocytes. Furthermore, ovariectomies can reduce some of these sex differences, while estrogen replacement can restore them, as seen in female rodents [33].

Understanding these interactions is crucial for developing potential therapies for neurological disorders, especially those with known sex differences in incidence or progression. However, it is important to note that the effects of estrogen on astrocytes can be complex and context-dependent, varying based on factors like brain region and age.

## 5. Sex Differences in Aging Astrocytes

**Figure 2:**
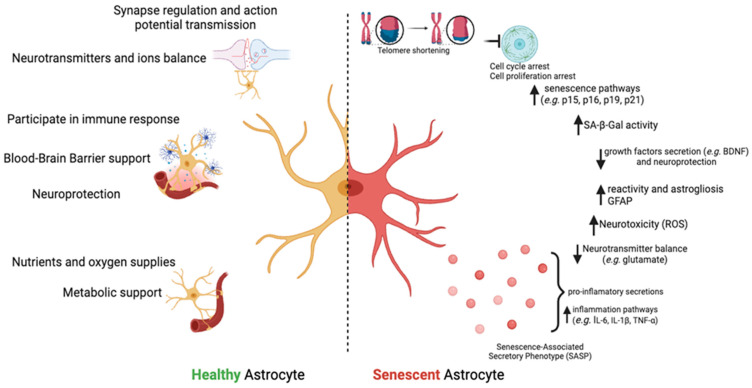
Differences between healthy and senescent astrocytes. Senescent astrocytes undergo cell cycle arrest accompanied by increased senescence markers such as p15, p16, p19, and p21 and increased β-galactosidase (SA-β-Gal) activity. In addition, they present Senescence-Associated Secretory Phenotype (SASP) by secreting pro-inflammatory molecules (e.g., IL-6, IL-1β, and TNF-α) and reveal higher levels of GFAP. Created with BioRender.com.

Astrocytes undergo cellular senescence like every other cell, a common cell process strongly affiliated with aging characterized by changes in morphology, function, activity, intercellular communication, as well as DNA- and gene-related features. Aging astrocytes undergo senescence-associated morphological changes that are characterized by a reduced number and length of processes, resulting in diminished synaptic coverage and neuronal functional support. Alterations in the nuclear morphology, including the depletion or total loss of major cellular components such as Lamin-B1 [68,69], have also been observed. Some common cellular senescence markers are cyclin-dependent kinase inhibitors (CDKIs), such as p16^INK4a^ and p21^Cip1/Waf1^ [70,71,72], which become upregulated and contribute to cell cycle arrest at the G1 phase. As a result, cells cease to divide and stay in a permanent growth arrest state, a hallmark of senescence [73,74,75]. Aged astrocytes usually also show increased acidic senescence-associated β-galactosidase (SA-β-Gal) activity due to increased lysosomal activity caused by accumulated cellular waste, mitochondrial dysfunction, or the activation of specific signaling pathways [73]. Moreover, they exhibit a pro-inflammatory phenotype (SASP) [76,77] and release more pro-inflammatory molecules, such as chemokines and cytokines. Although both sexes experience cellular senescence, studies have found that female mice are more prone to it than males [70,78]. This dimorphism was found in astrocytes as well. In mice, female astrocytes undergo more cellular senescence and accumulate higher levels of SA-β-Gal, p16^INK4a^, and p21^Cip1/Waf1^ in response to stress [31,32,34]. Furthermore, the CDKIs mentioned were reported to prevent DNA damage. Indeed, male astrocytes in mice accumulate more DNA damage markers with age than female astrocytes, possibly also due to differences in DNA repair efficiency and hormones [79] (Figure 2). These changes impair neuronal function by inducing oxidative toxicity, an imbalance of neurotransmitters recycling in the synaptic space, and a decrease in neuroprotection.

As the aging female approaches menopause, estrogen levels begin to plummet, which directly impairs the estrogen-driven neuronal glucose metabolism and oxidative phosphorylation [80]. Next, the endocrinological transition ultimately drives the aging female brain toward anaerobic glycolysis, thereby activating microglia, which start releasing proinflammatory cytokines, ultimately activating the astrocytes [80]. Another response to this neural starvation includes astrocytic activation to generate an alternative source of food, which they provide by breaking down white matter into lipids and ketones [81].

**Figure 3:**
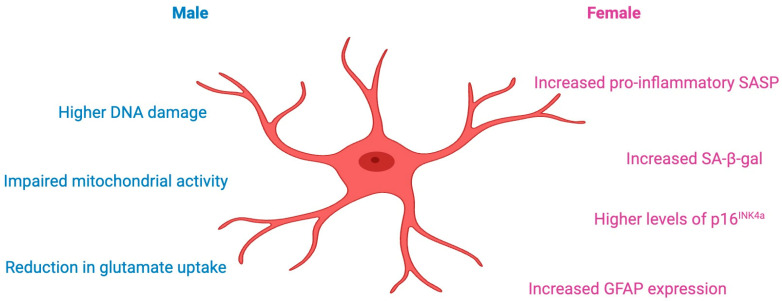
Comparative Profile of the Aging Male and Female Astrocyte. Male astrocytes exhibit higher rates of DNA damage and altered mitochondrial activity, suggesting an increased vulnerability to oxidative stress. In contrast, female astrocytes exhibit increased secretion of pro-inflammatory factors associated with the senescent phenotype (SASP), as well as increased β-galactosidase (SA-β-gal) activity and p16^INK4a^ levels, all classical markers of cellular aging. Increased expression of GFAP suggests greater glial reactivity in females. Created with BioRender.com.

The expression of the GFAP increases more significantly in female astrocytes in mice during aging [31,81]. Interestingly, female astrocytes appear to maintain a better mitochondrial function and energy metabolism with age compared to male astrocytes [82]. Male astrocytes tend to show a more pronounced age-related decline in certain functions, such as the glutamate uptake, compared to female astrocytes [83] (Figure 3).

## 6. Sex Differences in Astrocyte Response to Neurological Disorders

Hypoxia and ischemia [84] are common in neurological diseases. The ensuing cellular stress due toinsufficient oxygen levels and compromised blood flow, respectively, induces and accelerates alterations in astrocytic function that increase neuroinflammation and contribute to the severity of neurological disorders. Ischemic attacks and mortalities are significantly higher in men than women up until menopausal age. However, in the post-menopausal age, women are more affected than men [85]. Brain injury triggers astrocyte activation and the synthesis of vital neurotrophic factors that promote neuronal growth and recovery, such as Insulin-like Growth Factor 1 (IGF-1) and Vascular Endothelial Growth Factor (VEGF). IGF-1 is renowned for its neuroprotective effects post-ischemia, and IGF-1 expression levels in rats were found to be significantly lower in female astrocytes from the age of 10–12 m as compared to younger female astrocytes [24,86]. As part of its neuroprotective properties, estrogen promotes angiogenesis post-trauma via VEGF expression; thus, the depletion of estrogen in aging females causes a dramatic decrease in their VEGF expression. Besides the potential link between declining VEGF and IGF-1 levels and declining neuroprotective estrogen levels in the aging female astrocyte, estrogen could also have an age-dimorphic effect, being neuroprotective in young females and neurotoxic in aging females [87]. Even so, these effects appear to be female-specific: in rats, female astrocytic IGF-1 levels plummet with age while male levels remain constant, suggesting that astrocytic IGF-1 works in a sexually-dependent manner [24,33,88,89].

Following DNA damage, there is evidence that astrocytes in female mice have a greater tendency to undergo cellular senescence compared with male astrocytes, which tend to prefer apoptosis or malignant transformation [44]. Of note, sexual discrepancies are apparent in glioblastomas (GBM), which are 60% more likely to develop in males than females [43]. Mesenchymal glioblastoma (Mes-GBM), a subtype of GBM, causes the malignant transformation of astrocytes and deregulates cellular senescence proteins such as p53 and Retinoblastoma protein (Rb) that work to impede tumorigenesis. Male astrocytes exhibit a profoundly higher level of p53 and Rb inactivation compared to female astrocytes, which may support the explanation for the increase in their tumorigenesis in Mes-GBM [44]. Indeed, after administering cancer treatment, only the female astrocytes cooperated and stopped proliferating while expressing p21 and p16 senescence markers. Alternatively, the male astrocytes reacted completely differently to the treatment, assuming a full cancer cell phenotype with transformation and proliferation [31]. As mentioned previously, DNA damage repair declines with age, but less so in females than in male astrocytes. Perhaps estrogen may play a part in this sexual bias by inducing astrocytic senescence [29,32]. Estrogen-induced senescence may cause for an initially higher expression of senescent markers in female astrocytes due to accumulating estrogen throughout the years conjoined with their impaired ability to repair stressed cells compared to males.

Many neurological disorders and aging promote astrocyte activation, which allows for the release of pro-inflammatory factors that create an inflammatory environment [90]. Interestingly, a study testing the astrocytic response to chronic stress and inflammation revealed that while male astrocytes tend to secrete higher levels of proinflammatory cytokines [23], the female astrocytes tend to be more resistant to stress and display higher levels of ramifications [91]. Many neurological disorders or stress generate neural oxidative stress, characterized by the proliferation of reactive oxygen species (ROS). This oxidative stress boosts astrocytic dysfunction while ROSs amplify astrocytic pro-inflammatory secretion, thereby creating a deleterious feedback loop of an ongoing stress-induced secretion of detrimental factors by a malfunctioning astrocyte. In stress-induced murine astrocytes, increased ROS production was found in male astrocytes while superoxide ion levels in the mitochondria were increased in female astrocytes [92]. Of note, it was reported that human astrocytes exhibit increased sensitivity to stress compared to mouse astrocytes [93]. Along with ROS, viral infections can also trigger astrocytic activation and neuroinflammation by amplifying inflammatory cascades and pathological processes associated with neurological diseases [94,95]. Moving on, a notable increase was recorded in the NLR Family Pyrin Domain Containing 2 (NLRP2) DNA methylation in human astrocytes in response to viral infections, which may be more prominent in females than in males [94]. This expression may be linked to inflammasome activation within astrocytes. Moreover, astrocytes play a major role in regulating glutamate homeostasis and their dysfunction can lead to glutamate excitotoxicity that contributes to neuronal damage (a hallmark of various neurological conditions) [96]. Of note, it was reported that male astrocytes in rats exhibit increased impairment in glutamate uptake with age [97]. Metabolic stress has been observed in the context of neurological disorders such as Amyotrophic lateral sclerosis (ALS), Alzheimer’s (AD), Parkinson’s disease (PD), and Frontotemporal Dementia (FTD) and contributes to the astrocytic inflammatory profile that triggers neuroinflammation [98].

Nearly ⅔ of AD patients are women, with the ε4 allele of apolipoprotein E (ApoE4) being the most prominent genetic risk factor for developing late-onset AD (LOAD). Astrocytes have significant roles in maintaining neural homeostasis that are affiliated with AD such as producing ApoE and depositing it to the CNS, transporting cholesterol and other lipids between neurons, and clearing Amyloid β (Aβ) plaques that form sticky aggregates found in AD. While astrocytes attempt to clear Aβ, the ApoE4 variant is associated with increased Aβ plaque aggregation, which exacerbates AD pathology, especially in females [99]. Female ApoE4 astrocytes exert the weakest Aβ plaque coverage abilities, leading to an elevated Aβ plaque area and higher levels of neurite injury when compared with male ApoE3 astrocytes in mice [30].

Men are 1.5 times more prone to developing Parkinson’s Disease (PD) than women [100]. Research on sex differences in the astrocyte activity specific to PD is still limited. Female astrocytes in PD models generally show greater neuroprotective capabilities than male astrocytes. This may be partly due to E2 effects, which can enhance astrocyte-mediated neuroprotection [101]. Of note, female astrocytes appear to be more efficient in response to oxidative stress and its complications in neurodegenerative diseases [101]. In a study focused on the sexual bias of PD, the injection of -methyl-4-phenyl-1,2,3,6-tetrahydropyridine (MPTP) caused an increase in the GFAP mRNA levels only at the early stages in murine males, compared to females who displayed an increase in both the early and late time frames. However, late-stage GFAP mRNA levels may contribute to late-stage astrogliosis, and when coupled with estrogen, there is a higher neuroprotection of the dopaminergic neurons in PD [102]. In astrocytes, estrogen affects the production of GFAP via an estrogen-response element (ERE) placed in the GFAP promoter region [67]. Thus, the astrocytes partake in compensatory pathways in PD once regional neural death commences. These differences may partly explain the observed sex disparities in PD incidence and progression, with men generally at higher risk. 

Elevated GFAP levels in the bloodstream have been posited to correlate with various brain pathologies, among them AD [103,104,105]. Of note, a higher elevation of blood GFAP was reported in women compared to men [106]. This finding may be linked to the overall increased susceptibility of women than men to the disease. The neurodegenerative pathophysiology also includes an increase in senescent cells which release inflammatory cytokines, interleukins, and harmful growth factors to their environment [107,108]. Thus, learning about the astrocytes’ response to these stimuli is essential to understanding how they undergo cellular senescence. Therefore, it was found that in response to inflammation triggered by lipopolysaccharide (LPS) stimulation, male astrocytes in mice expressed significantly higher mRNA levels of pro-inflammatory cytokines IL6, TNF-α, and IL1β in vivo. In contrast, when protein levels were quantified via ELISA in vitro, female astrocytes secreted higher levels of proinflammatory TNF-α and lower levels of anti-inflammatory IL-10 than male astrocytes [23]. The intricate relationship between astrocytes, hormones, and their role in response to neuroinflammation is shifting to modern clinical applications. It was suggested that ERβ insertion into astrocytes decreased the hippocampal cognitive decline and atrophy in female mice [28]. Interestingly, another study found that ERα-bearing astrocytes are the primary neuroprotectors in response to inflammation in the CNS [109]. Both these studies, unrelated to each other, checked the neuronal and astrocytic capabilities in ER pathways. Surprisingly, both studies found that only the ER-bearing astrocytes, not the neurons, had a significant neuroprotective effect. Nevertheless, it was reported that postmenopausal female astrocytes produce estrogen in the form of estrone (E1), rather than estradiol (E2), of whom the latter has the preferential affinity for the ERα receptor [110,111,112,113]. Different forms of estrogen may attribute to the differences in estrogen activity in the aging female astrocyte. 

In Multiple Sclerosis (MS) studies, murine male astrocytes exhibited increased astrogliosis and secretion of Macrophage Inhibitory Factor 1 (MIF-1), a proinflammatory cytokine imperative to the astrocytic response post-injury and neuroinflammation [114]. Interestingly enough, female astrocytes may prove to be better candidates for MS recovery, with more effective remyelination abilities [114]. Therefore, MS treatments may bring about differing results in male versus female astrocytes, potentially influencing the treatment efficacy as a whole in a sex-dependent manner.

## 7. Conclusions

Sex differences in astrocytes can be observed from morphology to gene expression and activity. Due to their essential role in maintaining brain homeostasis, pathological changes in astrocyte activity can contribute to the development of neurological diseases. Understanding pathways that link to these changes can lead to developing novel strategies for personalized medicine in neurodegenerative diseases. 

## Figures and Tables

**Table 1:** Sex-Based Differences on Astrocyte Activity and Function.

Activity and Function	Brain Region	Male/Female	Human/Animal Model	Age (Pups/Adults)	Reference
Higher mRNA expression of inflammatory cytokine	Cortex	F	MO	Pups	[23]
Astrocytic glutamate clearance decreases with age	Cortex	F	R		[24]
Astrocytic MIP-1 production increases with age	Cortex	F	R		[24]
Increase in GFAP expression	Diencephalon	F	R	Pups	[25]
Increase in GFAP expression in DG and CA1 regions	Hippocampus	F	MO	Adults	[26]
Increases in astrocytic mGluR3 enhance memory	Hippocampus	F	MO	Mid-aged	[27]
ERβ treatment alleviates hippocampal neuroinflammation and cognitive impairment in GDX midlife female astrocytes	Hippocampus	F	MO	Adults	[28]
Aging causes increase in senescence markers and decrease in DNA damage repair	Hypothalamus	F	MO	Adults	[29]
APEO4 astrocytes have a deficiency amyloid plaque coverage		F	MO	Adults	[30]
Expression of TSPO in response to LPS		F	MO	Pups	[23]
Higher levels of p16 (cellular senescence) in response to stress		F	MO		[31]
Higher levels of astrocytic cellular senescence		F	MO		[32]
GBM astrocytes undergo more cellular senescence and less transformation		F	MO and H	Adults and pups	[31]
Astrocytic IGF-1 levels decrease with age		F	R	Adults and pups	[33]
Release more IL-10 compared to during acute inflammation		F	R	Pups	[34]
Female midlife ERβ-KO astrocytes have worse cognitive skills, more regional atrophy, and more DAM compared to ERβ-KO neurons	Hippocampus	M and F	MO	Adults	[28]
2567 differentially regulated genes between female and male GBM astrocytes		M and F	MO	Pups	[31]
105 differentially regulated genes	Cortex	M and F	H	All	[35]
7 upregulated genes and 23 downregulated genes	Hemisphere +cerebellum	M and F	MO	Pups	[36]
Increased astrocyte presence, complexity, and differentiation	ARC (hypothalamus)	M	R	Pups	[37]
Earlier developmental increase in astrocytic RNA expression	Cortex	M	MO	Pups	[38]
Higher mitochondrial maximal respiration rate	Cortex	M	R	Pups	[39]
More TSP-2-promoted synaptogenesis	Cortex	M	R	Pups	[40]
Increased surface density of immunoreactive GFAP+	Hippocampus and globus pallidus	M	R	Adults	[41]
Increased astrocyte complexity	Left MePD	M	R	Adults	[42]
Increased astrocyte cell count	Right MePD	M	R	Adults	[42]
Sex-specific DNA damage responses in astrocytes contribute to the higher glioblastoma risk		M	H	Adults and children	[43]
Earlier increase in GFAP mRNA levels upon MPTP intoxication		M	MO	Adults	[44]
Astrocytes have increased amyloid plaque coverage and reduced neurite damage		M	MO	Adults	[30]
Higher levels of Rb inactivation in male Mes-GBM astrocytes than females		M	MO and H	Adults and pups	[44]
Astrocytic IGF-1 levels remain constant with age		M	R	Adults and pups	[33]
Produce more TNFα compared during acute inflammation		M	R	Pups	[34]

Abbreviations from Table 1: F—Female; M—Male; MO—Mouse (model); R—Rat (model); H—Human (model); MIP-1—Macrophage Inflammatory Protein 1; GFAP—Glial Fibrillary Acidic Protein; DG—Dentate Gyrus; CA1—Cornu Ammonis 1; mGluR3—Metabotropic glutamate receptor 3; Erβ—Estrogen Receptor Beta; GDX—Gonadectomy; APOE4—Apolipoprotein E4; TSPO—Translocator Protein; LPS—Lipopolysaccharide; IL-10—Interleukin-10; Erβ-KO—Estrogen Receptor Beta- Knock Out; GBM—Mesenchymal Glioblastoma Multiforme; IGF-1—Insulin-like Growth Factor 1; DAM—Disease-Associated Microglia; ARC—Arcuate Nucleus; TSP-2—Thrombospondin-2; MePD—Medial Posterodorsal (amygdala); MPTP—1-Methyl-4-phenyl-1,2,3,6-tetrahdropyridine; Rb—Retinoblastoma Protein; TNFα—Tumor Necrosis Factor Alpha.
